# Action-based confidence sharing and collective decision making

**DOI:** 10.1016/j.isci.2024.111006

**Published:** 2024-09-20

**Authors:** Nicolas Coucke, Mary Katherine Heinrich, Marco Dorigo, Axel Cleeremans

**Affiliations:** 1 Center for Research in Cognition and Neurosciences, Université libre de Bruxelles, Brussels, Belgium; 2 IRIDIA, Université libre de Bruxelles, Brussels, Belgium; 3 Moral and Social Brain Lab, Department of Experimental Psychology, Universiteit Gent, Ghent, Belgium

**Keywords:** Cognitive neuroscience, Social sciences, Psychology

## Abstract

Joint action research explores how multiple humans can coordinate their movements to achieve common goals. When there is uncertainty about the joint goal, individuals need to integrate their perceptual information of the environment to collaboratively determine their new goal. To ensure that a group reaches a consensus about the goal, collective decision making among the individuals is required. Collective decision making can be facilitated by nonverbal expressions of opinions and associated confidence levels. Here, we show that confidence sharing in groups of 2, 3, and 4 individuals can be studied using their trajectories when jointly moving toward one of several options. We found that both opinions and confidence levels can be distinguished in individual movement trajectories, and found that movement features can predict an individual’s influence. Our results suggest that movement trajectories are a valid way to study confidence sharing in human collective decision making.

## Highlights

• Groups of 2, 3, and 4 human participants make movement-based consensus decisions
• Opinions and confidence levels are visible in movement trajectories
• Movement features predict an individual’s influence on the decision
• Movement trajectories reflect opinion dynamics

## Graphical abstract

**Figure:**
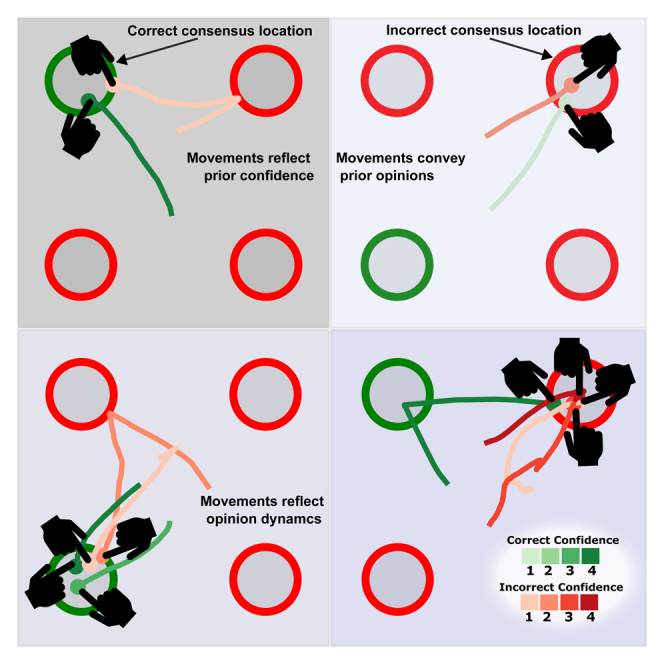

## Introduction

Joint action typically involves interpersonal coordination of movements toward one shared direction or spatial location. This coordination can be facilitated by, for instance, shared representations about the task, environment, or action constraints.^1^^,^^2^ When the goal location is already agreed upon by the individuals involved, they only require minimal sensorimotor information exchange to successfully execute the action. For instance, two people carrying a large object out of a building can perform this joint action without explicit communication, provided they both know the exit location.

However, if they were to carry the object to a room at an unknown location in an unfamiliar building, they would not yet have a shared representation of their environment. They would have to reach an agreement on their goal destination based on their instantaneous observations of the building’s interior. In this example and other common joint actions, relying solely on verbal communication is often impractical or too slow.^3^ By contrast, nonverbal interactions (for example, through haptic interfacing) can lead to faster agreements about perceptual information without a decrease in accuracy.^4^

When engaged in a joint action, individuals can modulate the movements required for the joint action in a way that provides additional information to their partner, so that they can reach nonverbal agreements that are *action-based*.^3^ Previous studies have focused on an informed “leader” that could guide the movements of a naive “follower” to a specific location.^5^^,^^6^ For example, one of the two individuals carrying the object might be familiar with their shared environment and could guide the naive individual toward a target location.

In many real-world situations, there are no fixed distinctions between informed and uninformed individuals. For example, when both individuals jointly carrying an object are unfamiliar with their environment, they might acquire perceptual information differently while not knowing how (dis)similar their information is to that of their partner. In such cases, individuals can aim to infer each other’s preferred target locations rather than communicate explicitly about acquired perceptual information. Previous experiments with haptically coupled individuals have indeed shown that coordinated movements can be improved when the individuals are continuously engaged in predicting each other’s motor goals.^7^^,^^8^ This finding also holds for larger groups: in groups of three or four, individuals could enhance coordination by estimating the joint movement goal of the group.^9^

However, when the goal of joint action is to choose and jointly move to one of several possible locations in a shared environment, the mechanisms required are not yet fully understood. Efficient coordination in such situations requires a joint *commitment* to one option rather than another. Such a commitment can be achieved by means of a *collective decision-making* process that takes into account the perceptual evidence that is distributed among group members. In contrast to scenarios often studied in joint action research, a collective decision does not merely involve an informed individual signaling to an uninformed one; it requires individuals with initially disparate opinions to resolve their disagreements and reach a consensus. We view such a decision-making process (i.e., collectively deciding on and jointly moving to a location in a shared environment) as a distinct kind of joint action that has not yet been extensively studied in humans. In one pertinent study, two participants integrated their perceptual information by converging as closely as possible to one target location in continuous space.^10^ However, only one target was in the environment; there were not several options to choose from.

A collective decision based on perceptual evidence is most likely to be accurate when each individual’s influence on the decision is proportional to the quality of their individual estimate.^11^ Experimental work with pairs of human participants has furthermore shown that two individuals can weigh each other’s opinions during decision making by sharing their self-assessed confidence levels as proxies for evidence quality.^12^ When using such weighting, the decisions depend on how accurately the pair’s members can calibrate their expressions of confidence and understand each other’s relative confidence levels.^13^ That is, individuals with similar quality of perceptual evidence in support of their opinions should express similar levels of confidence, and those with dissimilar evidence quality should express correspondingly higher or lower levels of confidence—however, humans have been shown to struggle with calibrating their relative expressions of confidence to relative evidence quality.^14^

Despite indications that humans can understand each other’s confidence levels through nonverbal expressions,^15^^,^^16^ confidence sharing in joint action has rarely been studied. It is not yet clear whether the speed and accuracy of joint actions could be improved by means of confidence sharing. Nevertheless, confidence-sharing mechanisms have been shown to be effective in collective decisions driven by ongoing haptic interactions,^4^ suggesting that such mechanisms would also be relevant for the physical coordination necessary in joint action. In decision making, confidence is often highly correlated with reaction times,^17^ and using the shortest reaction time as a heuristic for selecting the most accurate opinion has been shown to give similar results to selecting the opinion expressed with highest confidence.^18^ Furthermore, sharing reaction times has been shown to improve group accuracy in binary decisions: individuals with higher-quality information often form an opinion earlier and exert a larger influence on the group than individuals forming an opinion later.^19^^,^^20^

Besides reaction times, confidence can also be reflected in movement trajectories. In embodied individual decisions, movement is typically initiated before the individual’s internal decision-making process is complete^21^^,^^22^^,^^23^ and therefore *cognitive leakage* to the sensorimotor systems^24^ can result in aspects of the internal decision-making process being observable in the individual’s movements. In economic games, for example, kinematic cues can reflect the intention to act prosocially.^25^^,^^26^ In other decision-making contexts, movement trajectories can show the decision options supported by individuals over time and can show, for example, when a change of mind occurs.^27^^,^^28^ Movement-based interactions can also allow individuals to establish joint movement plans when they lack full information about each other’s movement goals.^29^^,^^30^ Interestingly, studies of movement-based group decision making have shown that a minority of informed individuals can steer a group decision by moving early and consistently in a certain direction.^31^^,^^32^ Although these collective decision-making studies have not yet included consideration of confidence levels, it is likely that group members interpreted the movements of others in terms of confidence; indeed, humans have been shown to accurately infer the goal of observed actions^33^ and the degree of confidence associated with it.^15^

In short, there is strong support in the literature for the idea that cues about the confidence levels of individuals could improve performance in joint action. In this article, we study the use of confidence sharing in joint action. In particular, we study a joint action scenario that combines movement to a common location with the decision-making process that selects the location.

**Figure 1:**
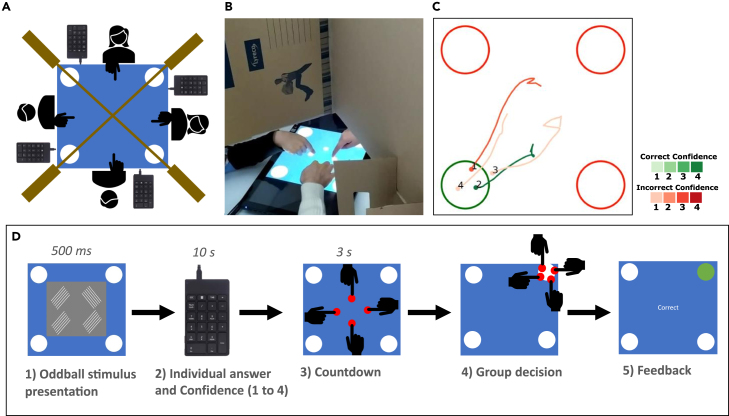
Experimental setup (A and B) Participants sat around a table with a touch screen. Each participant had a keypad (not visible to other participants) on which they could indicate their private answer and confidence level. Cardboard barriers were placed between participants such that only participants’ arms were visible to one another. Participants could drag circular avatars across the touch screen to one of four potential target locations. (C) Example of trajectory data of one trial with 4 participants. The color of the trajectory indicates whether the answer that the participant privately indicated on their keypad was correct. The color saturation of the trajectory reflects the participant’s privately indicated confidence level. The 4 large circles indicate the possible target locations. The correct target location is indicated in green. (D) A gray square is flashed on the screen containing four Gabor patches (illustrated as white gratings), one of which had a different orientation than the other three. Next, participants could privately indicate which one of the patches they thought deviated from the other three (and thus corresponded to the correct target location), and the confidence they had in that opinion. Then, participants placed their fingers on the circular avatars on the touch screen. A consensus decision is made when all participants have placed their avatars at the same potential target location. Afterward, feedback about the decision is shown on the screen.

We created a setup in which groups of 2, 3, or 4 participants could make a decision by jointly moving their index fingers to one of four target locations on a shared touchscreen (Figures 1A and 1B). Cardboard barriers were placed between participants so that the available social information was limited to observations of each other’s hand movements, without any verbal communication or facial expressions. Because our aim was to study true peer-to-peer interactions, we avoided a clear-cut distinction between informed and uninformed individuals. Rather, each participant observed the same difficult-to-distinguish stimuli, and any variation in their informedness, beyond stochastic variation, arose from differences in their perceptual competency. Specifically, at the start of each trial, the group was briefly shown a gray rectangle with four blurred Gabor patches, one of which had a different orientation than the other three, signifying that it corresponded to the correct target location (Figure 1D). After the stimuli presentation, participants privately indicated the answer they thought was correct, along with their confidence in their answer, before beginning the group decision phase.

Using the individual answers, individual confidence ratings, and movement data from the group decision phase, we tested several hypotheses explaining how the individuals pool their opinions during a movement-based group decision. The first hypothesis we tested is related to previous studies with pairs, which have shown that each individual’s influence on the joint decision is proportional to their respective confidence level.^4^^,^^18^ We tested the hypothesis that this finding would hold for groups of up to four individuals in our setup. Secondly, to study observable markers of opinion and confidence in movement trajectories, we assessed whether the more confident individuals would behave similarly to “informed individuals” in previous studies with large groups, by moving early and consistently.^32^^,^^34^ We also tested the closely related hypothesis that movement features found to indicate opinion and confidence would also predict participants’ influence during a trial. Thirdly, we studied whether the influence an individual has on the group would be predicted not only by their confidence level but also by the number of individuals advocating for the same opinion. The effects of majority and minority influence have been studied extensively in non-embodied decision making,^35^ but it remains an open question how minority and majority subgroups influence joint actions and movement dynamics. We tested the hypothesis that movement dynamics under majority influence versus minority influence would differ, in groups of three or four. In addition to testing these three hypotheses, we also completed an exploratory assessment motivated by the prior observation that humans can form metacognitive judgments based on the movements of others.^15^^,^^16^ We used several movement features to calculate measures of tacit metacognitive sensitivity^36^^,^^37^ and assessed whether those movement features are indicative of an individual’s likelihood to possess the correct opinion.

## Results

In each trial, participants attempted to select the uniquely oriented Gabor patch from a set of four, briefly flashed. The trials had four possible difficulty levels, based on the contrast of the Gabor patches (see STAR Methods), with 16 trials per difficulty level (a total of 64 trials).

In each trial, after observing the Gabor patches, each participant indicated a private decision and confidence level on their keypad, and then participated in a group decision. Group decisions were completed by participants unanimously moving their fingers to one of the four target locations on the touch screen.

### Group and individual accuracy

**Figure 2:**
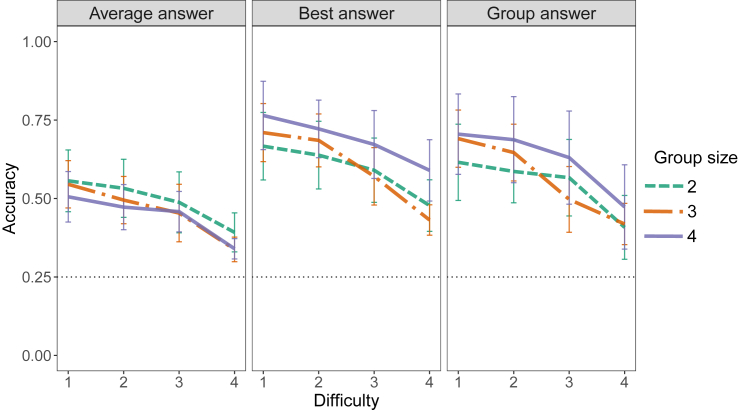
Accuracy of decision making for different levels of difficulty The different colors indicate different group sizes. Error bars indicate 95% confidence intervals. The dotted line at 0.25 indicates the accuracy levels associated with random answers. The different panels indicate four different kinds of decision accuracy calculated from the same data: (1) the average of private answers in the group, (2) the most accurate individual of each group, and (3) the consensus decision.

We first verified our experimental setup by assessing whether accuracy was meaningfully impacted by difficulty (see Figure 2). We defined individual accuracy as the percentage of trials in which the participant’s personal opinion prior to interaction was correct, and group accuracy as the percentage of trials in which the collective opinion of the group after interaction was correct.

We found that both average individual accuracy and group accuracy were negatively influenced by difficulty (individual estimate: β = −0.06, SD = 0.01, t = −9.66, *p* < 0.0001; group estimate: β = −0.08, SD = 0.01, t = −9.75, *p* < 0.0001) and positively influenced by training (individual estimate: β = 0.08, SD = 0.03, t = 2.20, *p* = 0.034; group estimate: β = 0.11, SD = 0.05, t = 2.15, *p* = 0.038). There was no significant effect of group size on the accuracy.

Furthermore, if participants can encode task-relevant information in their movements, then the group accuracy should be higher than the average accuracy of individuals. To assess this, we ran a model that included the type of opinion (individual or group) as a fixed effect and found that the accuracy of group decisions was more accurate than the average answer of individual decisions (β = 0.11, SD = 0.01, t = 8.99, *p* < 0.0001), indicating that, in our setup, the average participant can improve their performance by interacting with the others. However, the group accuracy is worse than the accuracy of the best-performing individual in the group (β = −0.05, SE = 0.01, t = −4.13, *p* < 0.0001), indicating that there is on average no collective benefit for the best group member. However, the group accuracy was better than the accuracy of the best individual in 11 out of 44 analyzed groups (2 groups of 2 participants, 4 groups of 3 participants, and 5 groups of 4 participants). Previous research on perceptual decision making with two individuals found that a positive collective benefit arose when the performance of individuals in the group was relatively similar.^4^^,^^12^^,^^18^ To assess whether this could explain why some groups in our data had this benefit and others had not, we quantified whether the variance in accuracy within one group could predict the collective benefit (group accuracy minus the accuracy of the best individual) for that group. Neither the variance in accuracy within one group (*p* = 0.29) nor the interaction with group size (*p* = 0.44) was significant predictor of the collective benefit (Figure S2).

Both the group accuracy (β = 0.076, SD = 0.018, *p* < 0.001) and the collective benefit (β = 0.061, SD = 0.016, *p* < 0.001) were significantly higher in the second part of the experiment, while the neither the average (*p* = 0.147) nor the best (*p* = 0.338) individual performance of each group improved from the first to the second half of an experimental session (Figures S3 and S4). This suggest that participants do not become better at the individual perceptual aspect of the task (i.e., discriminating the stimuli) but that they do improve their ability to share information and make decisions as a group.

### Confidence and accuracy determine influence within trials

**Figure 3:**
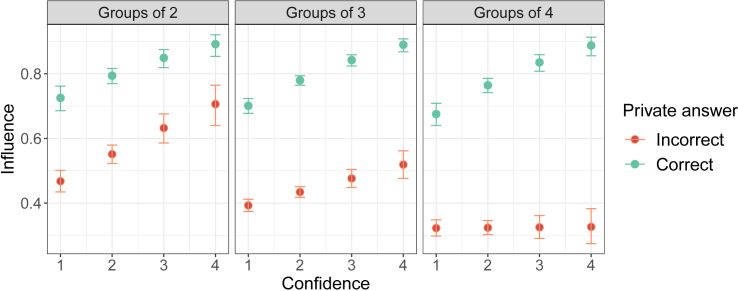
The relationship between influence and confidence The percentage of influence indicates the probability of a participant’s private answer ending up as the consensus decision. Dots represent means and error bars indicate 95% confidence intervals. The figure is produced by fitting an intercept for each level of confidence, as well as the interaction effects with correctness and number of participants. Correct private answers (green) resulted in more influence than incorrect private answers (orange). Higher indicated confidence levels resulted in higher influence. The different panels indicate different group sizes.

Next, we assessed whether the private answers and confidence levels of the participants meaningfully impacted the consensus decision. To do so, we ran a GLMM that predicted an individual’s influence on the consensus decision, given the correctness of the private answer and indicated confidence level. We defined ‘influence’ as a binary variable that becomes ‘1’ if an individual’s private opinion ends up as the consensus decision, and ‘0’ otherwise. Figure 3 shows predictions of the model for the probability with which an individual’s private answer would end up as the consensus decision, depending on whether that private answer was correct and the confidence level it was expressed with. The GLMM revealed that the probability of influencing the consensus decision increases for individuals that indicated the correct private answer (β = 1.08, SD = 0.45, *p* = 0.017) and level of confidence (β = 0.66, SD = 0.03, z = 4.65, *p* < 0.0001). Additionally, there was a negative interaction effect between the level of confidence and the group size (β = −0.16, SD = 0.04, z = −3.76, *p* < 0.0001), indicating that the effect of confidence on influence is less pronounced for larger groups. Lastly, a positive three-way interaction effect between confidence, correct, and group size (β = 0.20, SD = 0.07, z = 2.91, *p* = 0.004) indicated the decreased effectiveness of larger confidence in larger groups is most pronounced for incorrect individuals (see Figure 3).

### Confidence and opinions are reflected in movement trajectories

After establishing that the private answers and indicated confidence levels can influence consensus decisions, we asked whether the private answers and confidence levels are distinguishable in the individual movement patterns of participants during the collective decision-making process. To compare trajectories from participants starting movements at different locations, we first normalized trajectories so that each trajectory starts at distance ‘1’ from the target location, and becomes ‘0’ when the target location is reached. We pooled data across group sizes and considered the first 10 s of every trial. In trials shorter than 10 s, the final value of the trajectory was used for the remainder of the period (see STAR Methods). First, we calculated the *absolute* distance to and speed toward the potential target location indicated in the private answer, for individual trajectories. To investigate whether participants’ movements would become more informative when assessed in relation to the movements of the other participants in the same trial, we also calculated the distance to and speed toward the chosen target location *relative* to that of the other participants in the same trial.

### To the correct target location

**Figure 4:**
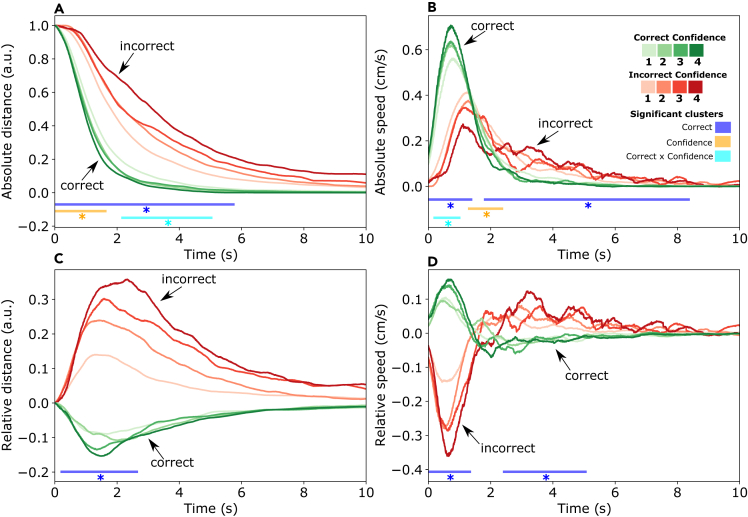
Movement trajectories with respect to the correct target location The color of each trajectory (green or red) indicates whether the private answer of an individual is correct or incorrect, and the shade of the color indicates the confidence level. Each line corresponds to the average of one type of trajectory for trials that ended in a correct consensus. The horizontal lines underneath the trajectories indicate significant clusters with a significant effect of being correct, confident, or an interaction. (A) Each trajectory represents the distance of the individual to the correct target location throughout the trial (normalized between the start and end position). (B) The speed of each participant in the direction of the correct target location. (C) The normalized distance to the correct target location relative to the other participants in the same trial. (D) The speed in the direction of the correct target location, relative to the other participants in the trial.

To isolate the effect of private answers and indicated confidence levels from that of the outcome of the consensus decision, we first looked only at trajectories where the consensus decision was correct. This ensures that every normalized distance trajectory starts at 1 (starting distance from the correct target location) and ends at zero (when arriving at the correct target location). Figure 4A shows that individuals with correct private answers approach the correct target location faster than those with incorrect private answers. High confidence amplifies the effect of opinion: confident correct individuals approach the correct target location the fastest, while confident incorrect individuals approach it the slowest. This is reflected in the results of the cluster permutation test, which reveal a significant cluster for the correctness of the private answer from 50 ms to 5740 ms, and a cluster for the interaction effect between confidence and correctness from 2170 ms to 5030 ms.

The speed of trajectories (Figure 4B), shows that correct individuals have a high initial speed toward the correct target location, while incorrect individuals move to the correct target location later and with a lower speed. There is a significant cluster for the correctness of the private answer at the beginning of the trial (0 ms–1380 ms) where correct individuals have the highest speed, and a cluster later in the trial (1820 ms–8360 ms) where incorrect individuals have the highest speed toward the correct target location. At the crossover of these two clusters (1310 ms–2370 ms) there is a significant effect for confidence since confident individuals (both with correct and incorrect private answers) temporarily have the lowest speed, possibly because the confident correct individuals have already arrived and the confident incorrect individuals still have to initiate their revision. At the beginning of the trial, there is a significant interaction effect where confident correct individuals have the largest speed and confident incorrect individuals have the lowest speed toward the correct target location.

The distance to the correct target location relative to the other participants in the same trial is shown in Figure 4C. The negative values for correct trajectories indicate that these are on average closer to the correct target location than the other individuals in the same trial. Incorrect participants are further away from the correct target location than other individuals in the same trial. This observation is supported by a significant cluster for the correctness of the private answer from 220 ms to 2650 ms. There was no significant effect of confidence.

The speed relative to the others in the trial is shown in Figure 4D. Initially, individuals with the correct private answer move more quickly (than others in the same trial) to the correct target location, while those with the incorrect private answer move slower. Later on in the trial, this effect seems to be reversed: correct individuals have the lowest relative speed while incorrect individuals have the highest relative speed.

### To the individually preferred target location

Above, we only took into account trials where the group converged on the correct target location. In order to assess the trajectory in all trials regardless of whether the group will converge on the correct target location, we converted trajectories to the distance and speed relative to each participant’s preferred target location, i.e., the location indicated in the private answer.

**Figure 5:**
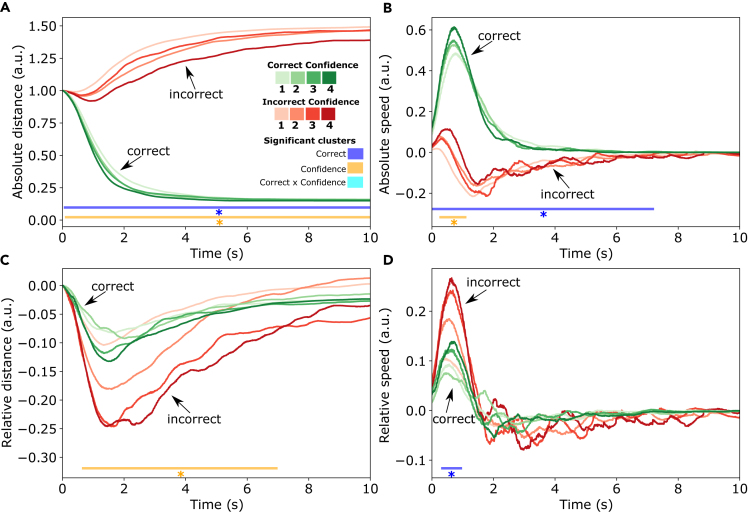
Movement trajectories with respect to the preferred target location The trajectories of each category are averaged across all trials (including those ending in an incorrect consensus). The color codes of the trajectories are similar to Figure 4. (A) Each trajectory represents the distance of the individual to their preferred target location throughout the trial (normalized between the start and end position). (B) The speed of each participant in the direction of their preferred target location. (C) The normalized distance to their preferred target location relative to the other participants in the same trial. (D) The speed in the direction of their preferred target location, relative to the other participants in the trial.

Figure 5A indicates that individuals with correct private answers arrive closer to their preferred target location than those with incorrect private answers, with a significant effect for the complete length of the trial. Note that groups are on average more likely to converge on correct than on incorrect consensus decisions, resulting in a larger distance from the preferred target location for incorrect individuals. A significant effect of confidence is also present, entailing that more confident individuals arrive more closely to their preferred target location (significant cluster from 90 ms to 10 s), irrespective of whether they have the correct private answer.

Figure 5B shows that both correct and incorrect individuals initially have positive speed toward their preferred target location and that higher confidence is associated with higher speed (significant cluster from 120 ms to 980 ms). However, the average speed of correct individuals is higher than that of incorrect individuals (significant cluster from 0 to 7.18 s). After 1 s, the speed of incorrect individuals becomes negative, indicating that incorrect individuals move away from their preferred target location as the trial progresses.

The relative distance plotted in Figure 5C shows that the most confident individuals move further away from the other group members toward their preferred direction (cluster from 670 ms to 6950 ms). Furthermore, visual inspection of Figure 5C indicates an overlap between trajectories that are confident and correct and those that are unconfident but incorrect, indicating that they are not easily distinguishable by the distance they move from the other group members.

Visual inspection of the relative speed toward the preferred direction shown in Figure 5D indicates that, initially, incorrect individuals have a higher relative speed toward their preferred answer (confirmed by a small significant cluster from 340 to 950 ms), while having a slightly lower speed later on in the trial. There also seems a clear effect of confidence in the initial speed, although none of these effects were confirmed by a significant cluster.

### Movement features predict influence within trials

**Figure 6:**
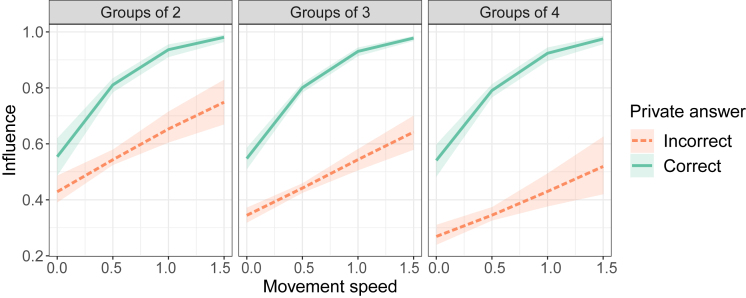
The relationship between influence and initial movement speed The percentage of influence indicates the probability of an individual’s private answer ending up as the consensus decision, as estimated by a GLMM. Shaded areas indicate 95% confidence intervals. Correct private answers (green) on average resulted in more influence than incorrect private answers (orange). Movements initiated with higher speed (assumed to be a tacit marker of confidence) resulted in higher influence. The different subplots indicate different group sizes.

After showing that confidence and opinions can be distinguished in movement trajectories, we asked whether these trajectories could, early on in the trial, predict whether an individual would be able to influence the consensus decision. As a simple marker of confidence in the trajectories, we took the absolute speed of the individual in the first second of each trial. We ran a GLMM similar to the one used for explicit confidence ratings (Figure 3). The model revealed a significant effect for movement speed (β = 1.12, SD = 0.47, z = 2.38, *p* = 0.017) while accounting for the effect of being correct, the average accuracy of the participant, and the effect of group size. A type II ANOVA analysis on the model terms showed that the variance in influence captured by the initial speed ($\chi^{2}\left(2\right)$ = 177, *p* < 0.0001) is smaller than the variance captured by whether the participant currently has the correct opinion ($\chi^{2}\left(2\right)$ = 527, *p* < 0.0001) and larger than that captured by the average accuracy of the individual ($\chi^{2}\left(2\right)$ = 109, *p* < 0.0001). This indicates that participants might use multiple sources of information to decide who to follow: both instantaneous movement features and long-term inferences about a person’s level of accuracy. Whereas the effect of explicit confidence ratings interacted with the group size and correctness (Figure 3), no significant interactions with the movement speeds were found here, indicating that the initial movement speed can be a good predictor of influence regardless of the situation (Figure 6). We also assessed two additional movement features: the speed variability of the movement and the onset time of the movement (Figure S5). Variability in movement speed could potentially be indicative of doubt, uncertainty, or second-guessing^26^^,^^38^ and we therefore would expect it to be negatively related to influence. We quantified the speed variability as the standard deviation of the movement speed in the first 2 s of the trial. We found that the main effect of the variability was not significant, but that there was a significant interaction effect of the speed variability with correctness in the current trial (β=46.8,SD=16.0,z=2.92,p=0.003). This indicates that speed variability might have different functions depending on the social context.

The onset time of the movement could be compared to reaction time (or deliberation time) and should therefore also be negatively related to perceived confidence,^15^ and likewise to influence.^19^^,^^20^ We quantified the onset time of movement as the time (in seconds) at which the individual moved at least 0.5 cm from the starting position. We indeed found that the onset time was negatively associated with influence (β=−0.43,SD=0.18,z=2.39,p=0.017).

To verify whether some participants had a decreased influence due to being physically obstructed by other participants (see Figure S6), we ran the GLMM again for the groups with 4 participants and added a coefficient for “obstruction” to the model that indicated whether a participant could be hindered by another participant in going to a potential target location. Neither the main effect nor the interaction effect were significant, indicating that participants were not hindered in exerting influence by physical interference in this setup.

### Majority and minority influence

**Figure 7:**
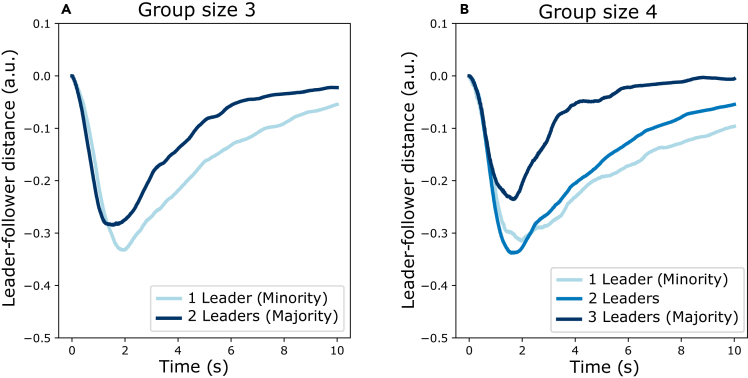
Majority and minority influence For each trial, the subgroup of leaders are identified as those individuals for which their private answer corresponded to the consensus decision at the end of the collective decision-making process. Here, only trials where participants disagreed are shown (i.e., not everyone has influence). The trials are grouped by the proportion of the group that were leaders. The averaged trajectory for each group of trials represents the relative distance that the leaders are closer to the consensus decision target location than the followers. Negative values indicate that leaders are closer than followers. (A) Averages of trials with 3 individuals. (B) Averages of trials with 4 individuals.

For groups with more than two individuals, whether and how someone can influence the group depends not only on their opinion or confidence but also on whether they are part of a majority or not. We identify majority influence as instances where more than half of the individuals in the group are able to impose the private answer they have in common on the whole group; we identify minority influence as instances where less than half of the individuals in a group are able to impose their private answer on the whole group. Majority influence occurred more often than minority influence in groups of 3 (resp. 46.20% and 34.50% of trials). For groups with 4 individuals, majority and minority influence were about equally prevalent (23.7% and 23.66% of trials). Most trials of groups with 4 individuals were guided by a subgroup of 2 (47.61% of trials). Figure 7 shows the differences in relative motion trajectories for participants in cases of either majority or minority influence. Visual inspection suggests that in trials with minority influence, the leading minority takes more distance from the rest of the group and the group reaches a consensus more slowly.

Indeed, a simple linear regression analysis on all trials with groups of 4 participants reveals a negative effect of the number of leaders on the consensus time (β = −1.31, SD = 0.31, t = −4.17, *p* < 0.0001) indicating that minorities take longer to impose their opinion on the group than majorities. We refer the interested reader to Figure S7 to see how the graphs in Figure 7 differ depending on whether the leading subgroup is correct and whether the leading subgroup is more or less confident than the follower subgroup.

### Metacognitive sensitivity of movement trajectories

**Figure 8:**
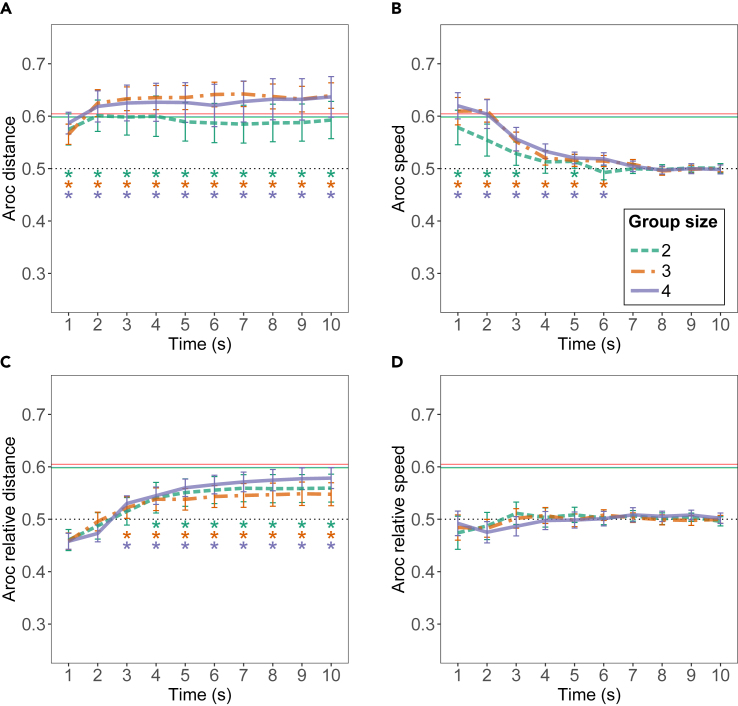
Metacognitive sensitivity based on movement trajectories The metacognitive sensitivity as the 2nd order area under the curve (Aroc) indicates how informative a trajectory at a specific time is of whether the individual has a correct opinion. The Aroc is calculated according to each of the 4 measures calculated from trajectories: (A) distance, (B) speed, (C) relative distance, and (D) relative speed toward the preferred target location. The Aroc is calculated using the averaged trajectory magnitude in a 1-s time window. The three different colored lines in each plot denote the average Aroc of the 3 group sizes. Error bars denote the 95% confidence interval. Colored asterisks denote whether the average Aroc for a group size at a particular time window is significantly above 0.5. Colored horizontal straight lines indicate the average Aroc calculated using the explicitly indicated confidence ratings for comparison.

Participants have to decide whether to follow other participants or not based on observed movements. For groups to come to accurate consensus decisions, the movements of individuals should thus be indicative of whether they have the correct opinion. In other words, movements should show some metacognitive sensitivity. We first assessed metacognitive sensitivity of each participant using the explicit confidence judgments that participants provided prior to the collective decision-making process. We used the second-order area under the curve (Aroc) to assess metacognitive sensitivity. False discovery rate corrected Wilcoxon ranksum tests confirmed that the Arocs for participants was significantly larger than 0.5 for all group sizes (2 participants: M = 0.60, SD = 0.08, *p* < 0.001; 3 participants: M = 0.60, SD = 0.09, *p* < 0.001; 4 participants: M = 0.59, SD = 0.10, *p* < 0.001) indicating that participants’ confidence judgments can be used to predict (better than random) whether their private answer was correct or incorrect, regardless of group size. Next, we assessed whether a measure of tacit confidence extracted from movements similarly allows determining whether an individual is likely to be correct. We calculated Aroc using each of the 4 measures calculated on trajectories as introduced in 3.3.2: distance, relative distance, speed, and relative speed to the preferred target location. For each measure, we calculated the Aroc in 10 different time windows throughout the trial for the 3 different group sizes (Figure 8).

The Aroc for absolute distance is not significantly influenced by time but increases with group size (β = 0.02, SD = 0.01, t = 2.83, *p* = 0.005), and is above 0.5 throughout the trial, indicating that distance to the preferred target location contains metacognitive information throughout the trial, especially for larger groups.

Aroc calculated from the speed decreases with time (β = 0.005, SD = 0.001, *p* < = 0.0007) and increases with the number of participants (β = 0.03, SD = 0.004, t = 6.86, *p* < 0.0001) and shows a negative interaction effect (β=0.003 SD = 0.0007, t = −5.12, *p* < 0.0001). The Aroc is above 0.5 for time windows 1, 2, 3, and 5 for 2 participants, and up to 6 s for groups of 3 and 4, indicating that initial speed contains metacognitive information, especially for large groups.

Aroc calculated from relative distances increases with time (β = 0.007, SE = 0.002, t = 3.81, *p* < 0.0001) but is not significantly modulated by group size. The Aroc is above 0.5 starting from the 3 s after trial initiation for group sizes 3 and 4 and starting from the 4 s for groups of size 2. Lastly, Aroc from the relative speed shows no effect of group size or time and is not significantly above 0.5 during the trial, possibly since there is too much variability in this measure.

To evaluate whether the tacit metacognitive sensitivity of trajectories reflects the explicit metacognitive sensitivity that participants indicated prior to the collective decision-making process, we conducted simple linear regression analyses with the tacit Aroc as a dependent variable and explicit Aroc and group size as independent variables. We conducted one regression for each type of tacit Aroc, where the Aroc for that measure is the average of time bins that were significantly above 0.5. This regression analysis revealed no significant relationship between the Aroc of any of the 4 measures and the Aroc calculated from explicitly indicated confidence measures.

## Discussion

To investigate how humans convey opinions and confidence in the context of joint actions, we created a new experimental design for collective decision making in small groups, in which participants select one of four potential target locations based on hard-to-distinguish perceptual stimuli. Participants’ private answers about the perceptual stimuli were clearly visible in their movement trajectories. For example, in cases where the consensus decision was correct, individuals with the correct private answer approached the corresponding target location faster than the individuals with the incorrect private answer. The relative distance between individual trajectories in the same trials reflects the opinion dynamics during the collective decision-making process; participants with different opinions initially move away from one another toward their preferred target location, which results in a peak in the relative distance. Next, some individuals gradually change their minds so that a consensus can emerge. Trajectory analysis has previously been used as a tool to track changes of mind in individual binary decision making.^27^^,^^39^ We here show that trajectory analysis can also be used to study the changes of mind in interactive collective decision making in small human groups.

The confidence that individuals indicated in their private answer was also apparent in the trajectories. Confident individuals moved more quickly toward their preferred target locations. This finding aligns with the positive relationship between short reaction times and high confidence found in previous research.^15^^,^^17^ Confident individuals were also slowest to revise their opinion when the location chosen by a majority of the other group members was different from their own preferred target location. This finding aligns with previous research showing that, the more confident an individual initially is, the less likely they are to change their opinion on their own^40^ or when receiving contradictory advice.^41^ The finding also aligns with the previous observation that individuals with better information are more adamant in executing their planned movements so as to guide others with less information.^30^

Apart from initial opinions and confidence levels, movements are also modulated by the desire to reach a consensus, which requires either to convince another person or to be convinced by someone else in case of disagreement. For example, low-confidence incorrect trajectories, on average, approach the preferred target location less often since they will less often be agreed to by others. Confident incorrect individuals have, on average, a smaller absolute speed to their preferred target location than correct individuals, but they move more in their preferred direction *relative to the others in the same trial* than a correct individual would do. In fact, both unconfident incorrect and confident correct individuals move at similar distances and speeds away from the group (as seen in Figures 4G and 4H), indicating that someone’s spatial distance from the group might be a sub-optimal way to assess their confidence level and the likelihood of them being correct.

This begs the question of whether someone uninformed of the correct target location could assess which individuals are likely to possess the correct information merely by observing their trajectory. The trajectory plots suggest that the trajectories that are most likely to be correct are those that approach their preferred target location quickly and without taking a great distance from the group. Individuals that are far from the group might be confident but the reluctance of others to follow them could indicate that they are not correct. The analysis of metacognitive sensitivity using trajectory data indicates that the initial absolute speed with which an individual moves toward their preferred target location and the absolute distance they are from that option during the trial can indicate whether this individual is likely to be correct. These trajectory measures seem to be most informative in larger groups, possibly since, in larger groups, incorrect individuals will more quickly be dissuaded from their initial movement direction, which will result in a less confident trajectory. Hence, one way to assess someone’s confidence or metacognitive ability could be the degree to which they are persuaded to move toward others’ preferences.

Surprisingly, the tacit metacognitive sensitivity derived from movement trajectories did not significantly correlate with the explicit metacognitive sensitivity calculated from the participant’s personal confidence ratings. This could suggest that the metacognitive evaluations made by participants during social interaction might be different from those based on perceptual information when responding individually.^42^ Additionally, the movement trajectories of decision-making processes could contain *ideomotor movements* that display parts of the cognitive process that the individual decision-maker cannot easily report explicitly.^43^ It has been proposed that dynamic social interactions may particularly elicit ideomotor movements, thereby revealing tacit knowledge and confidence.^44^ Our measures of tacit metacognitive sensitivity may capture these aspects, whereas explicit measures do not.

Influence was associated with faster movements at the beginning of the trial, which we took to be a tacit marker of confidence. This reflects the way that confident individuals could dominate a dyadic haptic collective decision-making process: by being the first to apply a significant force.^4^ Similarly, informed individuals in larger groups could influence the group by moving, first and consistently in a certain direction.^32^^,^^34^ The relation between movements and influence in our experiment was true across group sizes and was held for individuals with both correct and incorrect private answers. However, individuals with correct private answers had on average a higher influence for the same movement speed. We also quantified the variability in movement speed and expected this to be negatively associated with influence, since it can be a marker of uncertainty.^26^^,^^38^ We found that the effect of speed variability was indeed negative for incorrect individuals, but, surprisingly, was positive for correct individuals. For incorrect individuals (who usually hold opinions different from the majority), more variable movements might indicate uncertainty or second-guessing.^39^ Alternatively, for correct individuals, more variable movement speeds could potentially be signs of an accommodating leadership style, in which the leading individual trades off goal-oriented movements (confident movement toward the target) with socially-oriented movements (movements to ensure that others are following).^45^

In groups with more than two individuals, someone might not only be influenced by the movement features of others but also by the number of other individuals supporting a certain option. In many cases, individuals will use a majority rule and adopt the opinion most present in the group. Indeed, many of the trials with 3 and 4 individuals were completed under majority influence. However, there were also a large number of examples of minority influence, where a minority could influence a majority holding a different opinion. Differently from majority opinions, minority opinions can gain influence when the minority remains consistent in their opinion.^35^ In our experiment, we saw that trials with minority influence took longer to complete, which might be due to the time required for the minority to display consistency in their opinion. The minority’s persistence in moving to—and remaining at—a certain option could reflect confidence level. This behavior is similar to that observed in rodents, which wait longer at a certain location if they are more confident that a reward will appear there.^46^ Alternatively, minority influence can also be exerted by a confident individual who moves first, before the majority figures out that they have a majority.^32^^,^^34^ This suggests that, in continuous collective decision-making scenarios like ours, minority influence could arise in two complementary ways: either by moving strongly and early or by being very persistent.

An important question in collective decision making is whether the accuracy of the group is higher than that of the individuals within it. Our results show that the movement-based consensus decisions were on average more accurate than the private answers of individuals. However, group accuracy was not higher than the accuracy of the *best* individual in that group. Research on binary decisions made by dyads did result in a pair accuracy that surpassed the accuracy of the best individual—although this only occurred when the two had similar enough individual accuracies.^4^^,^^12^^,^^18^ In this study, we used a different paradigm and could not assess in the same way the ‘optimality’ of each group. However, the results point in the direction that the performance of groups in our task does not surpass the best individual. The sub-optimal performance of groups could indicate that, despite the observed presence of confidence weighting, making embodied decisions between four spatially distributed alternatives (with possible physical obstruction in two dimensions) poses a more challenging scenario for collective decision making than the binary decisions studied in previous research.^4^^,^^12^^,^^18^ Furthermore, the kinematic cues observed in our experiments might not convey confidence as effectively as either verbal communication or haptic interfacing, because the cues are not exclusively related to confidence. Movements made by a participant could be expressions of confidence, could be communicative actions intended to display preferences to others, or could be non-communicative movements made simply to advance toward a given decision option. Meanwhile, participants in Pezzulo et al.^4^ knew each other’s preferred answer before the interaction, because they only entered into the interaction phase if they had already disagreed on a binary choice, and thus the complete information channel could be used to exchange confidence levels. Future research could further investigate the relationship of movement features and group performance, for example by assessing whether the relative speeds of correct and incorrect individuals predict whether the group performance will surpass that of the best individual.

Group performance did not differ significantly with group size. Theoretically, larger groups are supposed to achieve greater accuracy when individuals are likely to be correct.^47^ The possible performance benefit of larger groups in our study might have been nullified by increased motor interference for larger groups,^48^ diffusion of responsibility,^49^ or because of herding.^50^^,^^51^

We assessed how participants’ prior confidence levels were reflected in the collective decision-making process; we did not register confidence judgments after the collective decision-making process has been completed. In future research, it might be interesting to investigate the evolution of individuals’ confidence judgments since it has been shown that social interaction can lead to more metacognitively sensitive confidence ratings^42^ but can also elicit irrational updates in confidence ratings caused by, e.g., confidence escalation (i.e., a boost in confidence that is not warranted by the information any of the individuals possess^52^^,^^53^).

Our study showed that the movement trajectories of people engaged in embodied collective decision making contain extensive data about their individual opinions, confidence, social influence, and interaction dynamics. Future study is needed to understand the conditions that would allow embodied collective decision making to optimally combine the information available to the individuals. Movement-based decision making is a promising and efficient way to simultaneously combine the guesses of many individuals, even on questions that are unrelated to spatial locations or physical actions.^54^ Understanding which features of movement adequately combine the relevant information is a crucial aspect in improving such collective decision-making processes. The information embedded in movements might also be of interest to roboticists studying embodied human-robot interactions^55^ or robot-robot interactions.^56^ For example, robots can time their actions to let human users infer the robots’ internal states such as task goals and confidence.^57^ Our research suggests that robots could also be designed to express their confidence in their movements in a way that allows them to make joint movement-based decisions with humans, even when the interacting robot and human have different levels of information quality. Our results could also be relevant for economic decision making, in which each outcome can be associated with a different payoff for each of the participants.^26^^,^^58^ Based on our results, it would be interesting to study whether people would be able to steer the group decision in their favor by moving early and consistently toward the option that is most rewarding for them.

### Limitations of the study

The main goal of this study was to assess how participants’ confidence levels are encoded in movements and thereby influence the collective decision-making process. To that end, we studied movement trajectories while participants were in interaction with others. A trajectory associated with a specific confidence level or private answer will thus not only reflect a participant’s individual opinion but also the behavior of the other group members. Since confidence could also be observed in movements made by individuals in isolation,^15^^,^^16^ it is not clear to what extent the appearance of confidence in movements in our paradigm was due to communicative intent.^59^ Future research could focus on distinguishing inherent markers of confidence and deliberate modulations of movements to communicate confidence. Future work could also investigate how individuals interpret one another’s movements to infer opinions and confidence levels, as well as the extent to which they might discount each other’s opinions during decision making.^60^ In our analysis, “influence” was a binary variable that quantified whether a participant’s initial private answer was the same as the consensus decision at the end of the trial. This measure abstracts from the actual movement dynamics through which participants could influence one another. Further research could investigate causal influence and leadership in motor trajectories, as has been done in many studies on sensorimotor coordination.^61^^,^^62^ Inferring leadership or influence in collective movements is quite challenging; it can depend on the context, individuals’ spatial position in the group, and interpersonal movement dynamics. Future work might look, for example, to the field of animal collective behavior where many sophisticated approaches are used to infer influence from movement data.^63^

## Resource availability

### Lead contact

Further information and any requests should be directed to and will be fulfilled by the lead contact, N.C. (nicolas.coucke@ugent.be).

### Materials availability

This study did not generate new unique reagents.

### Data and code availability

• De-identified participant data have been deposited at Zenodo and are publicly available as of the date of publication. The DOI is listed in the key resources table.
• All original code has been deposited at Github and is publicly available as of the date of publication. DOIs are listed in the key resources table.
• Any additional information required to reanalyze the data reported in this paper is available from the lead contact upon request.

## Acknowledgments

The authors would like to thank Ekaterina Kramskaya for helping with the data collection and Kobe Desender and Pietro Amerio for advice on the analysis. M.K.H., A.C., and M.D. acknowledge support from the F.R.S.-10.13039/501100002661FNRS, of which they are, respectively, postdoctoral researcher and research directors. N.C. was supported by the program of Concerted Research Actions (ARC) of the 10.13039/501100008367Université libre de Bruxelles and the 10.13039/501100008537Alice and David van Buuren Fund.

## Author contributions

Conceptualization, N.C. and A.C.; Software, N.C.; Investigation, N.C. and M.K.H.; Writing – Original Draft, N.C. and M.K.H.; Writing – Review and Editing, N.C., M.K.H., A.C., and M.D.; Funding Acquisition, A.C. and M.D.

## Declaration of interests

The authors declare no competing interests.

## STAR★Methods

### Key resources table

**Table:** 

REAGENT or RESOURCE	SOURCE	IDENTIFIER
**Deposited data**		

Experiment data	This paper	https://zenodo.org/records/10688060

**Software and algorithms**		

Task	This paper	https://github.com/NicolasCoucke/ActionBasedCollDMTask
Analysis	This paper	https://github.com/NicolasCoucke/ActionBasedCollDM

### Experimental model and study participant details

We recruited 158 participants (Mean age 19.54, SD = 3.19; 21 male). The participants were students from the Université Libre de Bruxelles who participated in the experiment in exchange for course credits. The study was approved by the ethical committee of the psychology faculty at Université Libre de Bruxelles. All participants provided informed consent before participating in the study. We did not analyze gender effects in this study, which might impact the generalizability of the reported findings.

### Method details

Participants were invited in groups of 2, 3, or 4 and sat around a table with an iiyama ProLite T2435MSC-B2 touchscreen on which a 29 cm by 29 cm square was illuminated. Participants’ bodies were hidden from one another by a cardboard barrier (Figure 1A). A space in the middle was cut out of the cardboard so that the participants could see the touchscreen and use their arms to interact with the setup (Figure 1B). Behind the barrier, each participant had a keypad on which they could indicate their individual answer. Each participant controlled a red avatar of 1.8 cm diameter and started the movement at one of four positions at 4.4 cm from the screen center. The potential target locations to choose from were four white circles of 8 cm diameter in the four corners of the arena, at 15 cm from the middle. A consensus decision (i.e., all group members favor the same option) was considered to be reached when all participants had moved their avatars to the same potential target location. When a consensus decision was reached, the decision-making process for that trial ended.

Each group of participants first conducted 4 tutorial trials, followed by a series of 64 experimental trials. At the beginning of each trial, there was a countdown of 5 s after which a gray box containing four Gabor patches (i.e., black and white sine wave gradient patterns shown through Gaussian windows) was flashed on the screen for 500 ms. Three of the patches were oriented in the same direction (either 45° to the left or 45° to the right) and one patch was oriented in the opposing direction, which denoted it as the correct target location. Participants had to detect which one of the patches deviated from the other three. Each trial consisted of one of four difficulty levels, determined by the Michelson contrast of the patches.^64^ In each session consisting of 64 trials, the trials were distributed across four different difficulty levels, with each level comprising 16 trials. These trials were interspersed throughout the session in a pseudorandom sequence, ensuring that trials of varying difficulties were evenly distributed, preventing clustering of the same difficulty level, and maintaining unpredictability in their order of appearance.

After the stimulus presentation, participants had a fixed time of 10 s to indicate their private answers (i.e., one of the four patches) and their confidence in their opinions (levels from 1 to 4) on their keypads. The time for indicating their private answer was kept fixed to 10 s and did not end early if all participants had answered, so as to not reveal any reaction time information. Then, one red avatar (red circle) per participant appeared in the middle of the screen. Avatars could not be moved from their starting positions until every participant placed their finger on their respective avatar. Participants then had to drag their avatar to one of the four potential target locations (white circles in the corners of the arena). When all participants placed their avatars at the same target location, thereby reaching a consensus decision, feedback was shown for 5 s until the next trial began.

### Quantification and statistical analysis

#### Sample size and data removal

We divided the participants into 18 groups of 2, 18 groups of 3, and 17 groups of 4 participants. We determined the number of participants based on well-known previous studies of collective decision making: up to 30 participants (15 groups of 2) for each of the experiments reported in Bahrami et al.^12^ and 36 participants (18 groups of 2) in Pezzulo et al.^4^ In our setup, we aimed to obtain a similar number of groups for each of the different group sizes. We recruited participants until the pool of students performing our experiment in exchange for course credits was exhausted. For the analysis, we removed trials in which at least one group member gave an invalid private answer (i.e., did not indicate an opinion and confidence rating within 10 s). For sessions in which at least one group member gave invalid private answers for more than half of the trials, we removed the full session. After the removals, we were left with 2816 total trials, with 17 groups of 2, 14 groups of 3, and 13 groups of 4 participants. Then, movement trajectories of all trials were visually inspected and trials with errant movement artifacts (e.g., sudden jumps in avatar position, avatar going out of arena) were removed (7 trials out of 2816). Note that, during the data collection, we also included 64 trials of a different experimental condition in which participants jointly controlled one large avatar. However, this condition resulted in a large number of movement artifacts and therefore was excluded from the analysis. The order in which the two conditions appeared was counterbalanced between sessions. The order in which conditions were presented is taken into account in the analysis as a training effect. This study was not preregistered.

#### Group and individual accuracy

To assess performance, we define an individual’s accuracy as the percentage of trials in which the individual had the correct private answer. Starting from individual accuracies, we could calculate for each session the average individual accuracy and the accuracy of the best individual in each group. Lastly, the actual group performance is the percentage of trials in which the group moved to the correct target location on the touch screen.

#### Statistical models

To assess factors impacting accuracy and influence, we used linear mixed models (LMM) and generalized linear mixed models (GLMM). We used the lme4 package in R^65^ for modeling.

To validate our setup, we first fitted two different LMMs to assess whether group and individual accuracy are modulated by the task difficulty in different trials. In the first model, we used individual accuracy as a dependent variable; difficulty (1–4), group size (2–4), and training (0 if the discarded condition came after the used data and 1 if it came before the used data) as fixed effects, and the session ID as a random effect:

(Equation 1) $${\text{accuracy}}_{i}=\beta_{0}+\beta_{1}\cdot {\text{difficulty}}_{i}+\beta_{2}\cdot \text{group}\_{\text{size}}_{i}+\beta_{3}\cdot {\text{training}}_{i}+u_{{\text{session}}_{i}}+\epsilon_{i}$$

The second model was identical except that group accuracy, instead of individual accuracy, was used as a dependent variable. Next, to determine whether the accuracy of the group consensus was higher than the average individual answer, we ran an LMM with accuracy as the dependent variable; kind (group consensus or average individual answer), difficulty, number of participants, and order as fixed effects and session ID as a random effect. Similarly, we ran one more LMM to compare the accuracy of the group consensus with the accuracy of the best individual in the group:

(Equation 2) $$\begin{array}{cc} a{\text{ccuracy}}_{i}= & \;\beta_{0}+\beta_{1}\cdot {\text{kind}}_{i}+\beta_{2}\cdot {\text{difficulty}}_{i}+\beta_{3}\cdot \text{group}\_{\text{size}}_{i}+\beta_{4}\cdot {\text{training}}_{i}+u_{{\text{session}}_{i}}+\epsilon_{i} \end{array}$$

To determine whether an individual’s private answer or confidence level could predict whether they would influence the consensus decision, we fitted a GLMM with a binomial distribution in which a participant was assigned an influence of 1 if the consensus decision matched their private answer and 0 if it did not. As fixed effects, we included the accuracy of the private answer (correct/incorrect), confidence (1–4), group size (2–4), and their interactions, as well as the average accuracy of the participant during the full session. The participant ID was used as a random effect:

(Equation 3) $${\text{influence}}_{i}=\beta_{0}+\beta_{1}\cdot {\text{confidence}}_{i}+\beta_{2}\cdot {\text{correct}}_{i}+\beta_{3}\cdot \text{group}\_{\text{size}}_{i}+\beta_{4}\cdot \left({\text{confidence}}_{i}\cdot {\text{correct}}_{i}\right)+\beta_{5}\cdot \left({\text{confidence}}_{i}\cdot \text{group}\_{\text{size}}_{i}\right)+\beta_{6}\cdot \left({\text{correct}}_{i}\cdot \text{group}\_{\text{size}}_{i}\right)+\beta_{7}\cdot \left({\text{confidence}}_{i}\cdot {\text{correct}}_{i}\cdot \text{group}\_{\text{size}}_{i}\right)+\beta_{8}\cdot \left(\text{average}\_{\text{accuracy}}_{i}\right)+u_{{\text{participant}}_{i}}+\epsilon_{i}$$

We included the average participant accuracy to account for the consideration that individuals might end up following a participant that has higher accuracy on average, regardless of that participant’s current movements. Additionally, we ran a similar model in which the individual’s movement speed at the beginning of the trial was used to indicate their confidence level (instead of using their self-reported confidence level). To assess whether participant’s influence could be affected by being physically obstructed by others, we also included a binary obstruction indicator as a fixed effect, which took the value 1 if another participant was partially obstructing their path to any of the potential target locations and 0 if not (see Figure S6 for details).

#### Movement trajectory analysis

We extracted the xy movement trajectories of all individuals in all trials and used several approaches to transform the 2D trajectories into 1-dimensional time series. For each trajectory, we calculated the Euclidean distance of the xy position of the participant over time to the correct target location in that trial. Then, to account for the different starting positions of the participants, we calculated the normalized Euclidean distance ${\hat{z}}^{\;\text{correct}}$, such that

(Equation 4) $${\hat{z}}_{i}^{\;\text{correct}}\left(t\right)=\frac{z_{i}^{\;\text{correct}}\left(t\right)}{z_{i}^{\;\text{correct}}\left(t_{0}\right)}\text{,}$$

where $z_{i}^{\;\text{correct}}\left(t\right)$ is the Euclidean distance to the correct target location at time *t* for participant *i*.

Additionally, we calculated the speed (rate of change $r_{\text{correct}}$) in the direction of the correct target location over time by taking the first derivative of the normalized Euclidean distance ${\hat{z}}^{\;\text{correct}}$ and smoothing the time series using a moving average filter with a window size of 0.5 s.

Then, because the collective decision-making process is interactive, we also calculated the distance and speed of an individual relative to the other individuals in the same trial. The relative distance for participant *i* (relative to other participants *j* in the same trial) is obtained by:

(Equation 5) $${\hat{z}}_{\;\text{rel},i}^{\;\text{correct}}\left(t\right)={\hat{z}}_{i}^{\;\text{correct}}\left(t\right)-\frac{1}{N-1}\sum_{i\neq j}{\hat{z}}_{j}^{\;\text{correct}}\left(t\right)\text{.}$$

The relative speed $r_{\;\text{rel},i}^{\;\text{correct}}$ is likewise calculated, using an equation analogous to that of Equation 5. This results in four 1-dimensional time series characterizing each participant’s movement trajectory: distance ${\hat{z}}_{i}^{\;\text{correct}}$, speed $r_{i}^{\;\text{correct}}$, relative distance ${\hat{z}}_{\text{rel},i}^{\;\text{correct}}$, and relative speed $r_{\text{rel},i}^{\;\text{correct}}$.

To assess each trajectory in terms of the participant’s individually preferred target location, regardless of whether the preferred target location was correct, we also calculated a second version of the four measures introduced above. With the same series of operations, but using the Euclidean distance from the position of participant *i* to the preferred target location of participant *i*, instead of to the correct target location. We thus obtain the distance ${\hat{z}}_{i}^{\text{preferred}}$, speed $r_{i}^{\text{preferred}}$, relative distance ${\hat{z}}_{\;\text{rel},i}^{\text{preferred}}$, and relative speed $r_{\text{rel},i}^{\text{preferred}}$. Note that ${\hat{z}}_{\;\text{rel},i}^{\text{preferred}}$ and $r_{\text{rel},i}^{\text{preferred}}$ are relative to the average distance/speed of other participants *j* in the same trial to the preferred target location of participant *i* (not of participant *j*). Overall, this results in eight different 1-dimensional time series to characterize each trajectory.

To assess whether movements reflect participants’ opinions and confidence levels, we grouped trajectories according to the private answer (correct/incorrect) and the indicated confidence level (1–4).

To contrast movement trajectories associated with different private answers and confidence levels, we conducted mass two-way ANOVA with cluster-based permutations to control for family-wise error rate (FWER).^66^ In EEG analysis, it is customary to use this technique to identify a cluster of samples in which there is an effect of an experimental condition. We divided the trajectories into eight “conditions” (1 per private answer–confidence level combination, pooled across all group sizes) and equalized the number of trajectories in conditions to end up with a balanced dataset with 132 trajectories per condition. We used the MNE-Python implementation of the cluster permutation test.^67^

Practically, the procedure worked as follows. First, we determined a threshold F-value corresponding to a *p*-value of 0.05 for the main effects of correct and confidence, and for their interaction effect. Next, we performed the cluster permutation procedure for each of the 3 effects. First, clusters of adjacent samples for which the F-value exceeds the threshold are identified in the original dataset. The F-values of all samples in the cluster are then summed to create a ‘cluster F-value’ that is saved as a test statistic for that cluster. Next, the condition labels of the dataset are scrambled 1000 times to create 1000 surrogate datasets. As for the original data, clusters of adjacent samples crossing the threshold F-value are identified in each surrogate dataset. Next, a distribution of all the cluster F-values that were discovered in the surrogate data is created. The *p*-value of a cluster in the original data is then determined by the percentage of values in the surrogate cluster distribution that are larger than the test statistic for the cluster of the original data. We only keep clusters that have a *p*-value below 0.05. By performing this procedure for each of the 3 effects, we can identify clusters for which there is a significant effect of correct, for confidence, and when there is an interaction effect. There was a large variety in the duration of trials (M = 3.71, SD = 3.67). We chose to analyze the trajectories in the first 10 s of each trial since 95.4% of trials were less than 10 s long. Trajectories of trials that were shorter than 10 s were padded at the end with the final value of the trajectory. We chose not to time-normalize the trajectories to prevent comparing events that take place over different timescales.

#### Majority and minority influence

We wanted to assess whether there are differences in majority and minority influence. We used a simple binary measure of influence for each trial; a person had influence (1) in a trial when their private answer corresponded to the consensus decision and no influence (0) when it did not. A consensus decision was said to have been made with majority influence if a majority (but not all) of the individuals’ private answers matched the consensus decision. A majority consisted of two individuals in groups of three, and three individuals in groups of four. A consensus decision was said to have been made with minority influence if one out of three or one out of four individuals’ private answers matched the consensus decision.

To assess how minorities and majorities could gain influence, we labeled the trajectories of the participants in each trial into leader trajectories (with influence 1) and follower trajectories (with influence 0). We then subtracted the mean distance to the potential target location chosen by the followers from that chosen by the leaders. Hence, the more negative this difference, the closer leaders are to the potential target location relative to followers. For the sake of comparison, we plotted this difference both for trials where the majority consisted of leaders and for trials where the majority consisted of followers.

#### Metacognitive sensitivity

We calculated the metacognitive sensitivity of participants’ judgments using the area under the receiver operator curve (Aroc).^68^ The Aroc allows assessing to what degree there are more true positives (high confidence for correct trials) than false positives (high confidence for incorrect trials) for any possible bias, that is, for the criterion that the participant uses to separate high from low confidence.^36^ In our case, with 4 levels of confidence, there are 3 possible criteria. According to the lowest criterion, a rating of 1 corresponds to low confidence while ratings 2–4 correspond to high confidence; for the second criterion, 1–2 correspond to low confidence and 3–4 to high confidence; for the highest criterion, 1–3 correspond to low confidence and 4 to high confidence. The receiver operator curve is constructed by drawing the true positive rate and the false positive rate for all three criteria. The Aroc is then defined as the area under this curve.

We first calculated the *explicit* metacognitive sensitivity by calculating Aroc using the confidence ratings (1–4) that individuals indicated individually. Next, we assessed whether the movement trajectories of participants could similarly show *tacit* metacognitive sensitivity. To assess at which time the trajectory contains the most information, we divided each trajectory into ten 1-s time windows and calculated the Aroc in each window. To calculate the Aroc for a certain time window, we took the mean value of a trajectory within the window for each trial. We then binned all the trial trajectory values per time window per participant into 4 equally spaced bins between the minimal and maximal value per window per participant. This resulted in a tacit confidence rating from 1 to 4 for each trial, which we used to calculate the tacit Aroc measure.

We used the Wilcoxon rank-sum test as a nonparametric way of assessing whether the average Aroc of all participants in groups of a certain size is above that of random answers. We used false discovery rate (FDR) correction on the *p*-values of this test to correct for performing the test for multiple group sizes and time windows. Lastly, we used simple linear regressions in R to assess how Aroc is modulated by the time windows and the group size.
