# Supplementary material for: Action-based confidence sharing and collective decision making
Source: iScience. 2024 Sep 20;27(10):111006. doi: 10.1016/j.isci.2024.111006 (PMC11490717; doi:10.1016/j.isci.2024.111006)
Supplement: Document S1. Figures S1–S7, Tables S1 and S2 [file mmc1.pdf]

iScience, Volume *27*

Supplemental information

Action-based confidence sharing

and collective decision making

Nicolas Coucke, Mary Katherine Heinrich, Marco Dorigo, and Axel Cleeremans

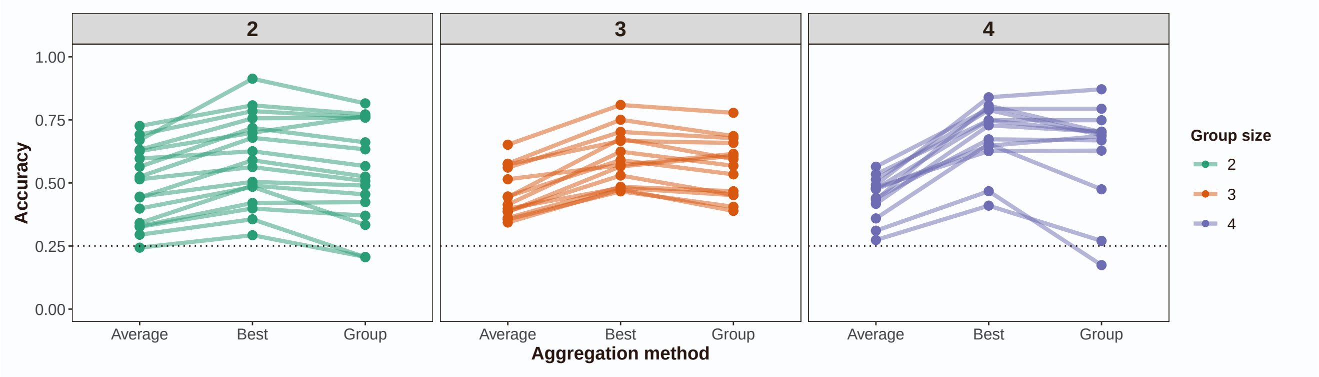

Figure S1. Accuracy of groups, Related to Figure 2. Each broken line represents the accuracy of one group. Each point in that line represents the group accuracy that is calculated through a different aggregation method. The first two are calculated based on the participants' private answers on their keypads, either 1) by taking the average accuracy of all group members, or 2) by taking the accuracy of the best group member. The third, labeled "Group", is the accuracy of the decisions made by the group on the touchscreen during the joint decision phase. After pooling difficulty levels, the average individual accuracy was 0.54 (SD = 0.04) for groups of two, 0.56 (SD = 0.01) for groups of three, and 0.60 (SD = 0.04) for groups of four. Meanwhile, the average group accuracy was 0.54 (SD = 0.20) for groups of two, 0.56 (SD = 0.12) for groups of three, and 0.62 (SD = 0.20) for groups of four. Despite the wide variety in accuracy levels, the group accuracy is higher than the average individual accuracy and slightly lower than the accuracy of the best individual (section 2.1 in the main paper).

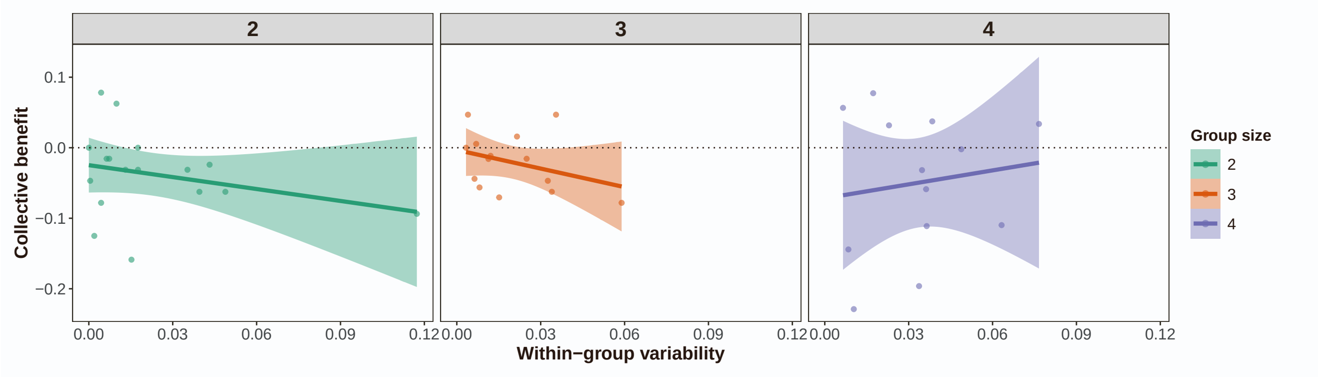

Figure S2. Association of the within-group variance of accuracy with the collective benefit, Related to Figure 2. Since we used a different experimental paradigm and analysis procedure, we could not calculate the accuracy or sensitivity (i.e., slope of the psychometric curve), which was used to quantify differences in individual performance in Bahrami et al.[1] Instead, we used the within-group variance of individual accuracies in each group as a proxy. The average within-group variance of participants' performance was 0.023 (SD = 0.029) for groups of two, 0.020 (SD = 0.016) for groups of three, and 0.033 (SD = 0.021) for groups of four. Results are reported in the main manuscript.

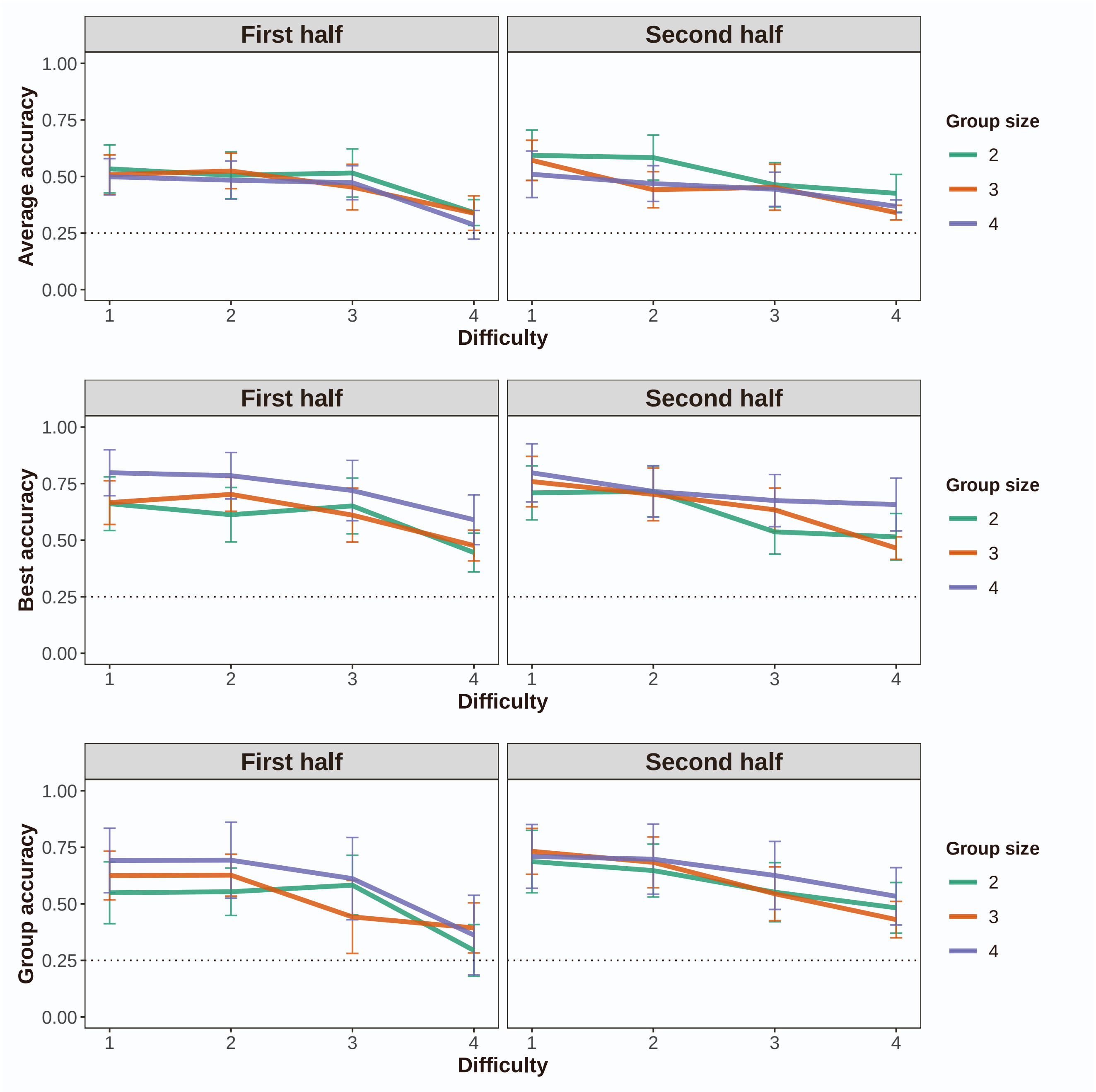

Figure S3. The effect of training on performance, Related to Figure 2. Decision accuracy in the first half (first 32 trials) and second half (last 32 trials) of the experiment, in terms of average individual accuracy (upper row), best individual accuracy in the group (middle row), and group decision accuracy (bottom row). Mean accuracy and 95% confidence intervals are given for each of the two halves (left and right columns), according to group size (color) and difficulty level (*x*-axis). For each group, we used a linear mixed model to assess whether there was any difference between the first and second halves of the sessions, in terms of average individual accuracy, accuracy of the best individual, or group accuracy. Results are report in the main manuscript.

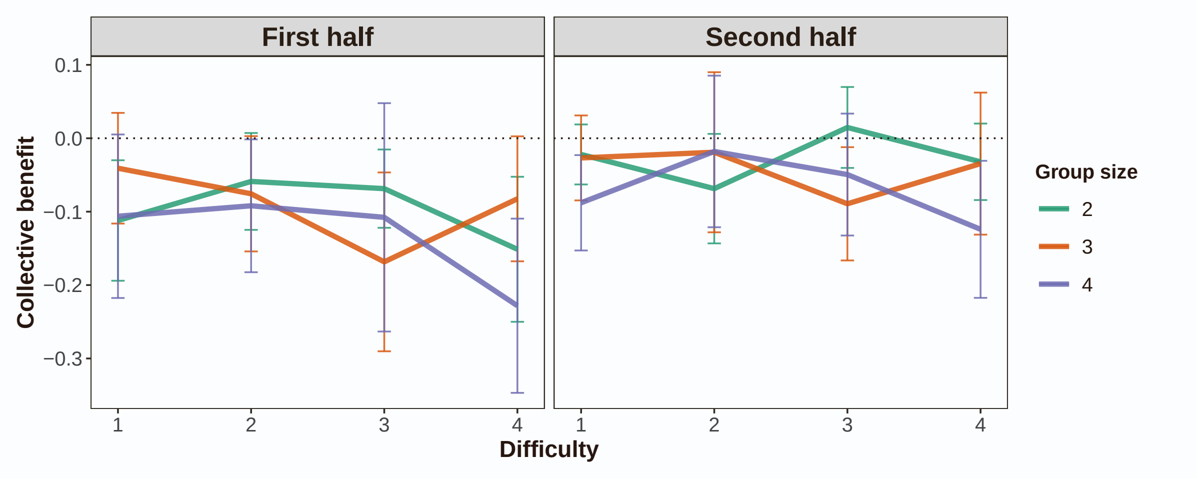

Figure S4. The effect of training on the collective benefit, Related to Figure 2. Collective benefit — i.e., the group performance minus performance of the best individual — in the first half (first 32 trials) and second half (last 32 trials) of the experiment. The figure shows the collective benefit for each of the two halves (left and right columns), according to group size (color) and difficulty level (x-axis). An improvement in collective benefit could indicate that improved group performance is because of groups learning how to better collaborate using their movements rather than individuals becoming better at perceiving the stimuli. Results are reported in the main manuscript.

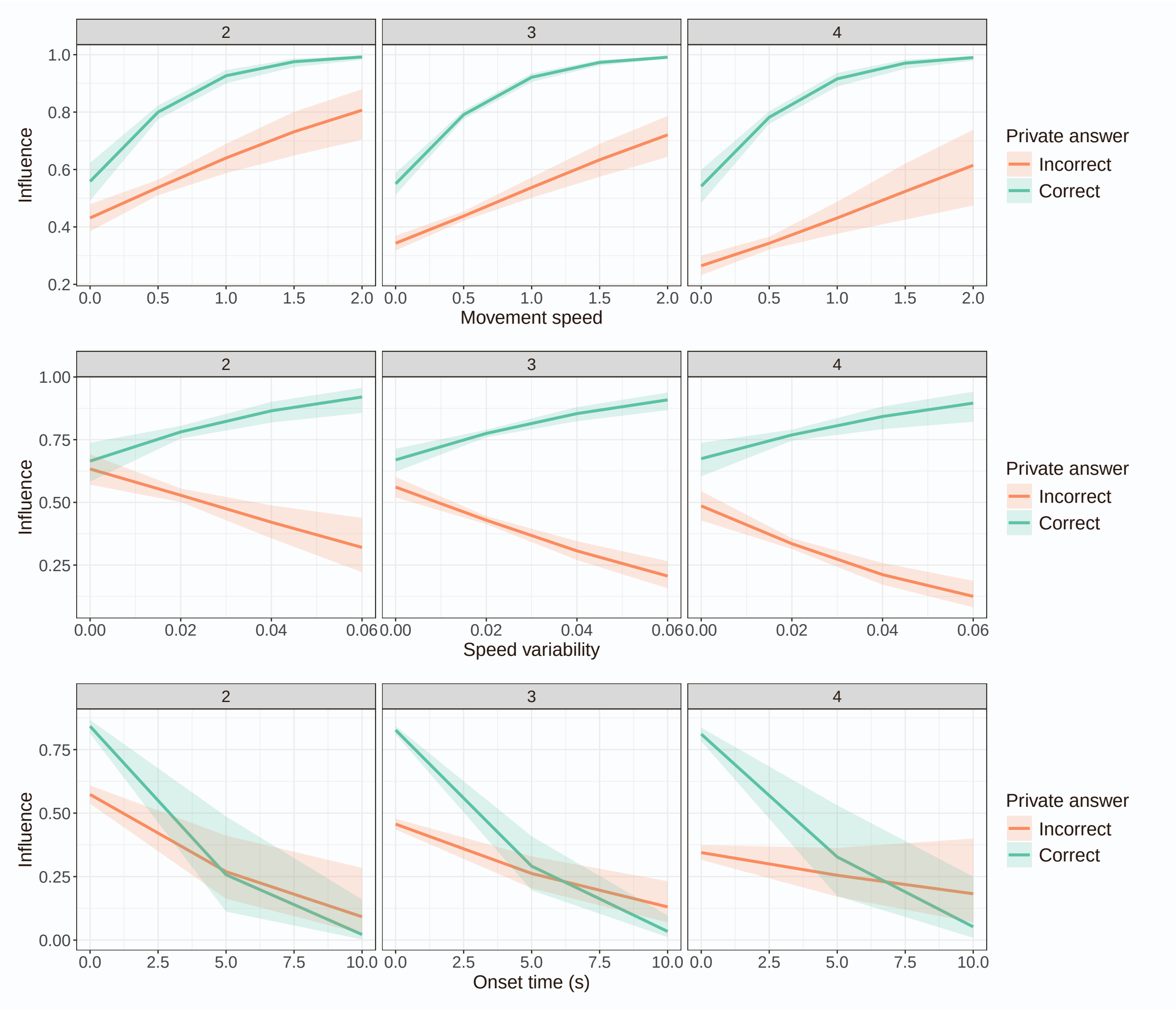

Figure S5. Influence predicted by different movement features, Related to Figure 6. The figure shows influence predicted by: initial movement speed (top row), speed variability (middle row), and movement onset time (bottom row). Columns represent group size.

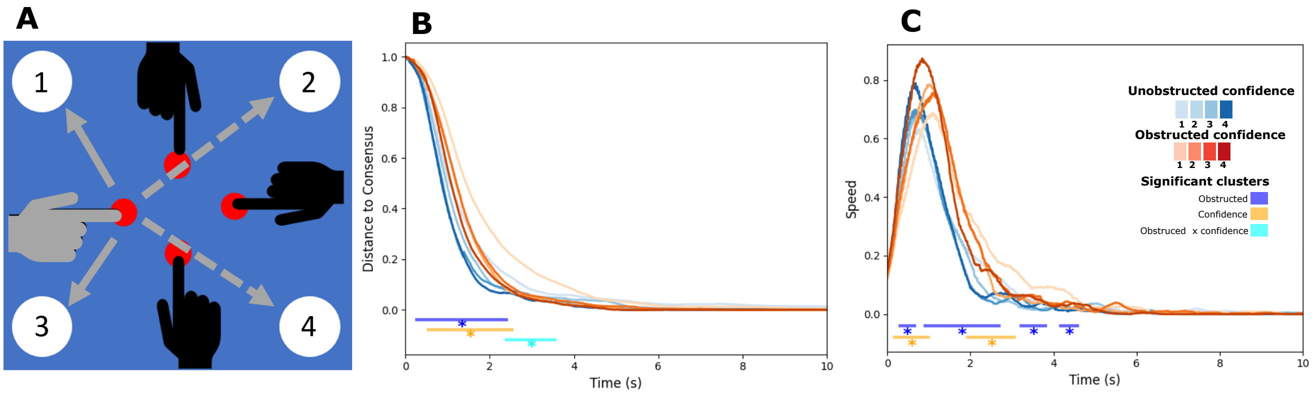

Figure S6. Movement interference, Related to Figure 4. A) As multiple participants are physically interacting on the screen, the ability of a participant to move in a certain direction can be obstructed by someone else. The focal participant, highlighted in grey, is more likely to be obstructed by the hands of other participants when attempting to reach targets 2 and 4 than when attempting to reach 1 and 3. Note that the options for which participants are most likely to be obstructed are also the ones for which participants had to move the furthest away from their starting position, which might also have influenced their movement trajectories. B) To evaluate to what degree movements are modulated by this physical interference, we assessed all trials with four individuals, splitting the trajectories into instances in which an individual's movement to the consensus decision was on their side of the screen (unobstructed) or on the opposite side (obstructed). Movement trajectories from correct trials, transformed to distance from the consensus option. The beginning of each trajectory is time-locked to the first movement of any individual in the trial. The color of each trajectory (blue or red) indicates whether or not they were possibly obstructed in moving toward their privately indicated answer. The shade of the color indicates the confidence with which that opinion has been indicated (darker for more confidence). The horizontal lines underneath the trajectories indicate for which periods the cluster permutation test indicated a significant modulation of trajectories according to obstructed/not obstructed (blue line) and confidence (yellow line). Unobstructed individuals approach the consensus location faster. C) Same as plot B, but with movement speed instead of movement trajectory. This revealed that obstructed individuals have higher speeds than unobstructed individuals. In section 4 of the main results, we investigated whether physical obstruction would affect an individual's ability to gain influence on the group decision by including a binary obstruction variable in a GLMM that predicted influence based on initial movement speed for groups of four individuals. Those result showed that the obstruction effect did not significantly affect the possibility of participants to influence the consensus decision.

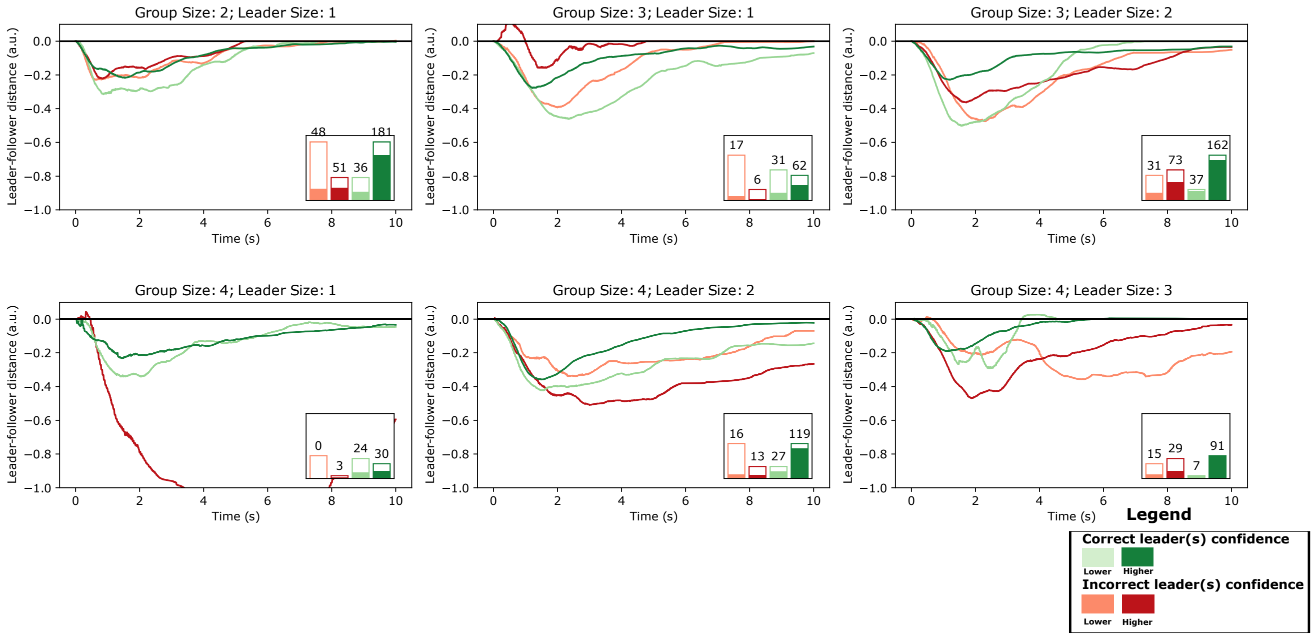

Figure S7. Interaction of majority and minority influence with opinion and confidence, Related to Figure 7. The way in which a consensus decision is influenced by a subgroup of individuals might differ depending on the opinion and relative confidence level of the subgroup. In this figure, each subplot depicts trials in which a leader subgroup of a certain size succeeded to convince the other subgroup to change their opinion to the leader subgroup's preferred target. Similarly to Figure 7 in the main paper, each plotted line reflects the average distance to the consensus location of the leader from the follower subgroups. The color of the trajectory indicates whether the leader subgroup had the correct or incorrect opinion. The shade of the color (light or dark) indicates whether the leader subgroup was privately either more (dark) or less (light) confident than the other subgroup. Each plotted line in a subplot is the average of all trials of a certain relative confidence/opinion combination for that subgroup size. The inset plots indicate how many trials are averaged into each line (full boxes) and how many total trials occurred with a subgroup of that size (that did or did not become leader subgroups).

		correct		confidence		interaction	
		cluster	p value	cluster	p value	cluster	p value
absolute	distance	50-5740 ms	0.001	50-1630 ms	0.034	2170-5030 ms	0.017
	speed	0-1380 ms<br>1820-8360 ms	0.001<br>0.001	1310-2370 ms	0.002	200-1000 ms	0.002
relative	distance	220-2650 ms	0.01	/	/	/	/
	speed	0-1340 ms<br>2430-5050 ms	0.001<br>0.001	/	/	/	/

Table 1. Cluster permutation results, Related to Figure 4. Detailed values for clusters found with the permutation test. Clusters and p values for trajectories with respect to the correct answer.

		correct		confidence		interaction	
		cluster	p value	cluster	p value	cluster	p value
absolute	distance	90-10000 ms	0.001	120-10000 ms	0.001	/	/
	speed	0-7180 ms	0.001	280-1090 ms	0.004	/	/
relative	distance	/	/	670-6950 ms	0.002	/	/
	speed	340-950 ms	0.01	/	/	/	/

Table 2. Cluster permutation results, Related to Figure 5. Clusters and p values for trajectories with respect to the privately preferred answer of the individual.

References

1. Bahrami, B. *et al.* Optimally interacting minds. *Science* **329**, 1081–1085 (2010).
